# Pamidronate therapy for hypercalcemia of childhood malignancy

**DOI:** 10.1186/1687-9856-2015-S1-P56

**Published:** 2015-04-28

**Authors:** Andrew Sng, Kah Yin Loke, Yvonne Lim, Cindy Ho

**Affiliations:** 1 National University Hospital, Singapore

## Background

Hypercalcemia is a common complication of adult malignancies, but uncommon in childhood cancers. The mechanism leading to hypercalcemia varies with the primary tumour and can include the following: 1) Increased osteoclastic activity at the site of the tumour in the bone marrow 2) A paraneoplastic phenomenon secondary to PTH-related peptide (PTHrP) secretion or 1,25–dihydroxyvitamin D by the tumour.

Patients with serum calcium above 3.5 mmol/L tend to be symptomatic and require prompt treatment. The symptoms are generally non-specific and include lethargy, nausea, vomiting, constipation and abdominal pain. Left untreated, hypercalcemia can progress to stupor and coma.

## Case Presentation

A 3-year-old girl presented with prolonged fever, hepatosplenomegaly, pancytopenia and multiple osteolytic lesions. She experienced significant weight loss of 5 kg over the past one month associated with anorexia and began to develop persistent daily fever up to 39.5°C with severe pruritus, constipation and irritability.

She was diagnosed to have Ebstein Barr Virus (EBV)-induced T-cell Lymphoproliferative disorder. However, she developed severe symptomatic hypercalcemia of 3.76 mmol/L associated with the paraneoplastic syndrome of an elevated PTH-related peptide, which was successfully treated with intravenous pamidronate (0.5 mg/kg administered as 2 doses), after conventional therapy with hydration and forced saline diuresis failed. Her serum calcium began to normalize to 2.3 mmol/L 2 days after pamidronate administration, with improvement in her symptoms of pruritus and temperament. She tolerated the infusion of pamidronate well with no adverse side effects.

## Conclusion

We have demonstrated the effective use of pamidronate as a single agent at a total dose of 1mg/kg to lower serum calcium levels in a patient with severe hypercalcemia of malignancy. In such cases of severe hypercalcemia, we recommend the early use of pamidronate since hydration with saline diuresis is unlikely to normalize serum calcium levels effectively.

Written informed consent was obtained from the patient for publication of this abstract and any accompanying images. A copy of the written consent is available for review by the Editor of this journal.

